# Immune Cell‐Mediated Retinoblastoma Development: Genetic and Molecular Mechanisms

**DOI:** 10.1155/ijog/6303747

**Published:** 2026-04-09

**Authors:** Xinyang Hu, Jingqiu Li, Jinhong Li, Wenxuan Chen, Haibo Zhang, Dan Li, Ming Zhu, Liang Pang, Siqi Fan, Suolang Yangzong, Ciren Deji, Dongwei Liu

**Affiliations:** ^1^ The First Affiliated Hospital of Anhui Medical University, Hefei, 230032, China, ahmu.edu.cn; ^2^ The Second Affiliated Hospital of Anhui Medical University, Hefei, 230601, China, ahmu.edu.cn; ^3^ School of Public Health of Anhui Medical University, Hefei, 230032, China; ^4^ Department of Ophthalmology, Shannan City People’s Hospital, Shannan, 856100, China; ^5^ Department of Ophthalmology, The Second Affiliated Hospital of Anhui Medical University, Hefei, 230601, China, ahmu.edu.cn

**Keywords:** IVs, Mendelian randomization analysis, RB1, retinoblastoma, single-cell RNA sequencing

## Abstract

Retinoblastoma (RB) is the most prevalent malignant tumor that originates within the eye in children, occurring at a frequency of around 1:20,000 to 1:15,000. RB typically occurs when both alleles of the retinoblastoma 1 (RB1) gene are deactivated. RB is associated with variations in the tumor suppressor gene RB1 and is inherited in an autosomal recessive manner, operating at the genetic level. Variation in RB1 occurs only when two alleles of the RB1 gene are mutated. The mechanism by which immune cells mediate RB1 to affect RB is still unclear. Using two‐sample Mendelian randomization (MR) analysis based on published genome‐wide association study (GWAS) summary statistics, we investigated the relationship between immune cells and RB1 proteins. Our goal was to understand their potential role in the development of RB in offspring. The analysis of alterations in RB tissue cells and potential molecular mechanisms was conducted using single‐cell RNA sequencing data. The research revealed the presence of 28 immune cells linked with RB1. These variables might potentially impact the likelihood of RB in children by altering the expression of the RB1 gene in their parents. Furthermore, we found differential gene expression in different cells of the RB tissue. EZH2, UBLCP1, and HKDC1 overlapped with the identified instrumental variables (IVs) of immune cells to investigate potential molecular mechanisms by which immune cells participate in RB processes.

## 1. Introduction

Retinoblastoma (RB) is a malignant tumor that is usually detected in individuals who are under the age of 5 [1]. It can develop during pregnancy, often caused by genetic mutations linked to malignant neuroplasmas and pineoblastomas. The disease has a global incidence of approximately 1:16,000–1:18,000 [2], and there are around 300 new cases reported annually in the USA [3]. There are regional variations in the incidence of RB, with higher rates observed in India. This could be attributed to a combination of genetic and environmental factors. The disease has an equal impact on both genders. In China, the incidence of RB is approximately between 1:20,000 and 1:15,000, which is consistent with the global rate. Around 1100 new cases are reported each year [2, 4, 5].

Significantly, a staggering 84% of Chinese patients get a diagnosis after their intraocular condition has already reached an advanced stage. This emphasizes the urgent need for early screening and heightened public awareness [6]. Understanding the development of RB involves studying the mutation of the retinoblastoma 1 (RB1) gene [2, 7], which plays a crucial role in regulating cell growth. When the RB1 gene undergoes a mutation, it leads to an abnormal increase in cell growth and differentiation, resulting in the formation of tumors [8]. Offspring who inherit the faulty RB1 gene have an almost certain 100% probability of developing RB [2, 9, 10]. In the dominantly inherited form, one mutation is passed down through the germinal cells, while the second mutation arises in somatic cells [11]. Using RB1 protein, we explored the connection between RB and immune cells.

Research indicates that the tumor cells of both late‐onset and early‐onset RB exhibit a striking resemblance to normal retinal progenitor cells. However, they do not display the differentiation markers typically associated with normal development [12]. In addition, there is a strong presence of immune gene expression in the early‐onset RB tumor cells, which leads to the buildup of various immune cells such as dendritic cells, monocytes, macrophages, and T‐lymphocytes within the RB tumors [12]. Studies have shown that post‐translational modifications of the RB1 protein may affect the behavior of immune cells. For example, the phosphorylation of RB1 can reduce its affinity for E2F1, thereby promoting entry into the cell cycle [13]. However, the exact causal relationship remains to be studied in depth. Mendelian randomization (MR) is a research technique that utilizes genetic diversity to study the causal influence of functions or phenotypes on disease outcomes, similar to randomized controlled trials (RCTs) [14, 15]. Unlike traditional RCTs, MR utilizes instrumental variables (IVs) to account for possible confounding factors without considering them as distinct interventions. IVs may be used in MR to estimate the causal influence of an exposure on an outcome when there is a clear cause‐and‐effect link between them [16]. This estimate is possible when the IV is linked to the exposure but is not affected by any confounding variables that may impact the connection between the exposure and the result. Crucially, it is essential that there be no direct cause‐and‐effect relationship between the IV and the result, except via the exposure. The estimate may be conducted using either a single IV or a collection of IVs for the exposure. MR has been extensively used across several disciplines and has yielded substantial outcomes. The Gene Expression Omnibus (GEO) database is a publicly accessible resource of gene expression data, offered by the National Center for Biotechnology Information (NCBI) in the USA. It serves as a repository for raw data obtained from microarray and other high‐throughput experimental methods [17]. Single‐cell RNA sequencing (scRNA‐seq) is a high‐throughput genomic technology that quantifies gene expression at the individual cell level, revealing cellular heterogeneity and changes in cell states during complex biological processes. The role of scRNA‐seq analysis is to provide unique gene expression profiles for each cell in a population, helping researchers identify different cell types, states, and functions, as well as their roles in health and disease. Our work used MR and scRNA‐seq data from GEO databases to investigate the underlying causal connections and potential processes linking immune cells and RB.

## 2. Materials and Methods

### 2.1. Study Design

**FIGURE 1:**
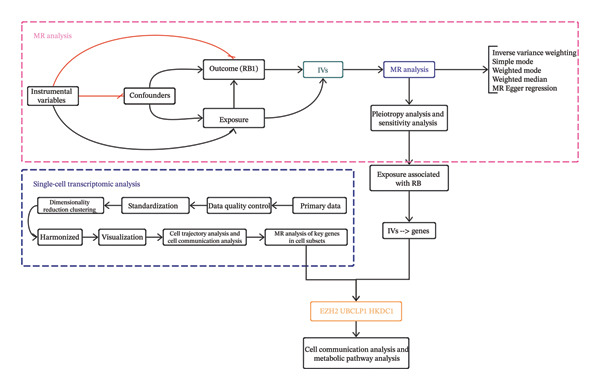
Summary of the main assumptions and methods used in MR analysis.

This research complied with the STROBE‐MR requirements and conformed to the fundamental principles of the Strengthening the Reporting of Observational Studies in Epidemiology (STROBE) recommendations [18]. The MR method is based on three assumptions: (1) It is crucial that the genetic variants used in the analysis show a strong correlation with the exposure being studied. (2) To ensure the accuracy of the analysis, the genetic variants selected as IVs for the exposure are completely unaffected by any potential confounding factors that may be linked to both the exposure and the outcome. (3) The research should not have the presence of horizontal pleiotropy, which refers to the situation where the IVs may only affect RB via exposure [16]. We conducted our study using publicly accessible GWAS summary information. No more data were gathered, and no further ethical clearance was necessary. Figure 1 depicts the method of doing research in a study.

### 2.2. Data Sources

#### 2.2.1. Circulating Immune Cells

GWAS summary statistics for each immune trait are publicly available from the GWAS Catalog (Accession Numbers from GCST0001391 to GCST0002121). A comprehensive analysis was conducted on a total of 731 immunophenotypes, which included several measurements such as absolute cell (AC) counts (*n* = 118), median fluorescence intensities (MFI) indicating surface antigen levels (*n* = 389), morphological parameters (MPs) (*n* = 32), and relative cell (RC) counts (*n* = 192). The MFI, AC, and RC features contain B cells, CDCs, mature T cells, monocytes, myeloid cells, TBNK (T cells, B cells, and natural killer cells), and Treg panels. The MP feature includes CDC and TBNK panels. The first genome‐wide association study (GWAS) research on immunological features included data from 3757 people of European descent, ensuring that there were no overlapping cohorts [19].

#### 2.2.2. Outcome Data Source

We obtained data on the RB1 protein from a GWAS that included 3301 European samples and 10,534,735 SNPs (prot‐a‐2496). These data came from a research on the genes encoding human plasma proteins, which found 1927 genetic relationships with 1478 proteins—a fourfold increase over what is already known—and 1104 trans associations with proteins.

### 2.3. MR Statistical Analysis

We used the inverse variance‐weighted (IVW) method as the main technique for MR analysis to assess the causal impacts of various exposures on RB1 protein. In addition, we used the MR Egger, weighted median, simple mode, and weighted mode methods. The objective was to examine the possible causal connection between these four exposure variables and RB by use of RB1 protein. The Wald ratio approach was used to determine the impacts of each SNP, while Cochrane’s *Q* test was used to evaluate the heterogeneity across the SNP instruments. If heterogeneity was discovered (*p* < 0.05), we used the random‐effects IVW test to get a conservative and robust estimator. The weighted median test yielded reliable results when at least 50% of the weights were from genuine IVs. The MR Egger regression test was used to address the issue of pleiotropy in over 50% of the IVs. The sensitivity analyses conducted in this study included the MR Egger intercept test, the global test for outliers (MR‐PRESSO), and the leave‐one‐out analysis. The MR Egger intercept and MR pleiotropy residual sum and outlier (MR‐PRESSO) global test were used to evaluate the presence of pleiotropy. The use of the leave‐one‐out methodology facilitated the identification of whether important outcomes were impacted by specific SNPs. The statistical analyses were conducted using *R* (version 4.3.3). The IVW, weighted median, simple mode, weighted mode, and MR Egger regression techniques were executed using the “TwoSampleMR” software (version 0.5.11). The MR‐PRESSO test was performed with the “MRPRESSO” software program. A significant criterion of *p* value < 0.05 was used.

### 2.4. scRNA‐seq Data Analysis

The scRNA‐seq data of RB (GSE174202) were acquired from the GEO database. The dataset comprises RB tissues generated by stem cells and RB patient tissues. The dataset used in this work has gained the required ethics clearances and is accessible to the public. The analysis of scRNA‐seq data was performed using the “Seurat” R program. Following the removal of low‐quality data, which consisted of genes expressed in less than three individual cells, cells expressing less than 1000 genes, and cells with mitochondrial gene content above 20%, we continued with further analysis. Afterward, we used the “NormalizeData” function to do the “LogNormalize” normalization on the data and then converted it into a Seurat object. The “FindVariableFeatures” algorithm was used to identify the top 2500 genes that exhibit high variability. Subsequently, the “RunPCA” function was used to conduct principal component analysis (PCA) on the genes with high variability, specifically focusing on the top 15 principal components (PCs). The “FindNeighbors” and “FindClusters” functions were used to conduct cell clustering analysis. The Uniform Manifold Approximation and Projection (UMAP) algorithm was then applied using the “RunUMAP” function, and cell clustering tests were carried out based on the UMAP‐1 and UMAP‐2 dimensions. In order to assign cell types to the cell clusters, we used the “SingleR” R program and conducted cell annotation using the Human Primary Cell Atlas as the reference dataset.

### 2.5. Key Marker Gene eQTL and RB1 Protein’s MR Analysis

The preparation of gene expression data included normalization, reduction of batch effects, and management of missing values. Distinctive marker genes for each cell type (progenitor cells, plasmablasts, and naive CD8 T cells belonging to neurons; plasmacytoid dendritic cells, plasmablasts, and progenitor cells belonging to neuroepithelial cells) were discovered in contrast to other cells. An eQTL analysis was conducted for each marker gene of the cell type. The conversion of gene symbols to ENSEMBL IDs was performed to maintain data consistency. SNPs of poor quality were eliminated, and genotyping fitting was performed. A strict criterion for the eQTL *p* value, such as 5 × 10^−8^, was established. SNPs associated with the main marker gene were extracted as IVs for MR analysis from the “finn‐b‐C3_NSCLC_ADENO_EXALLC” GWAS dataset. After performing *R* ^2^ and *F*‐statistic calculations for each SNP, only the SNPs of high quality were retained. The outcome data associated with these instrumental factors enabled the use of MR analysis using the TwoSampleMR program. We represented the data using volcanic maps and tree diagrams.

### 2.6. Seeking Genes Associated With Exposure

We accessed the internet database of the National Center for Biotechnology Information (NCBI) (https://www.ncbi.nlm.nih.gov/SNP/) to map each IVs to its closest gene. The found genes were compared with important marker genes that may have a causal association with the RB1 protein, serving as key genes.

### 2.7. Exploring the Function of Exposure Factors at the Single‐Cell Level

Initially, we conducted single‐cell RNA expression analysis to investigate the expression of the target gene at the individual cell level. We used visualization tools such as DotPlot and FeaturePlot to exhibit the expression patterns of important genes. The scMetabolism tool was used in metabolic analysis to assess the metabolic activity of macrophages. We used the DotPlot of metabolic pathways to illustrate the variations in cellular activity across distinct gene groups within certain metabolic pathways.

## 3. Result

### 3.1. Mendelian Randomization Analysis

Following quality control measures including LD effects and palindromic analysis, we found SNPs to be IVs associated with exposures for RB we have been working on (with the threshold of *p* < 1 × 10^−5^). Their details are shown in the supporting information (Supporting Table 1).

**FIGURE 2:**
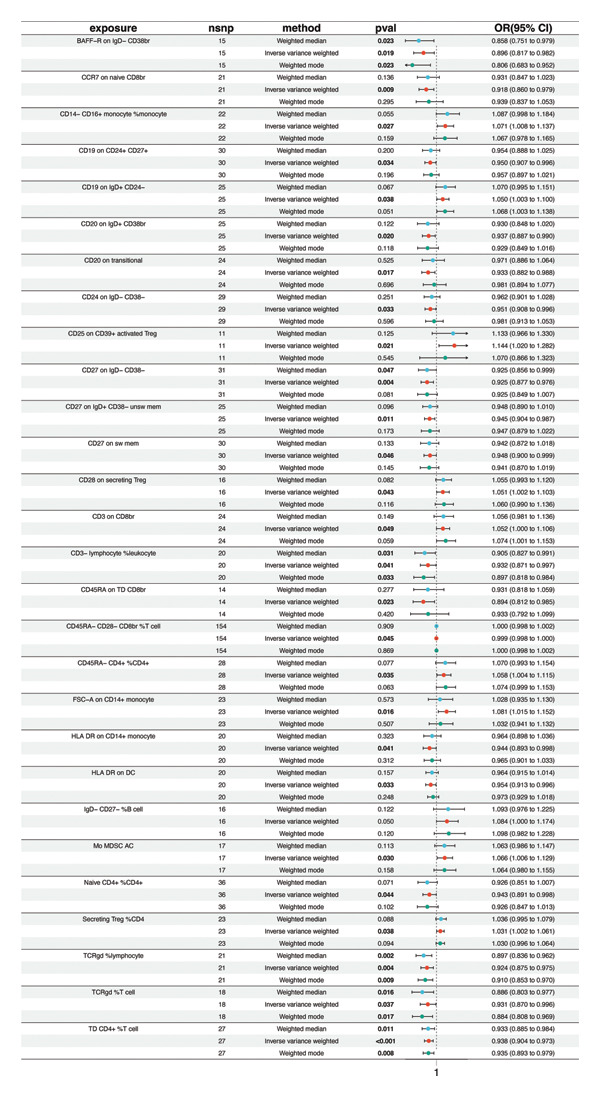
MR results of immune cells with a causal relationship to RB1.

**FIGURE 4:**
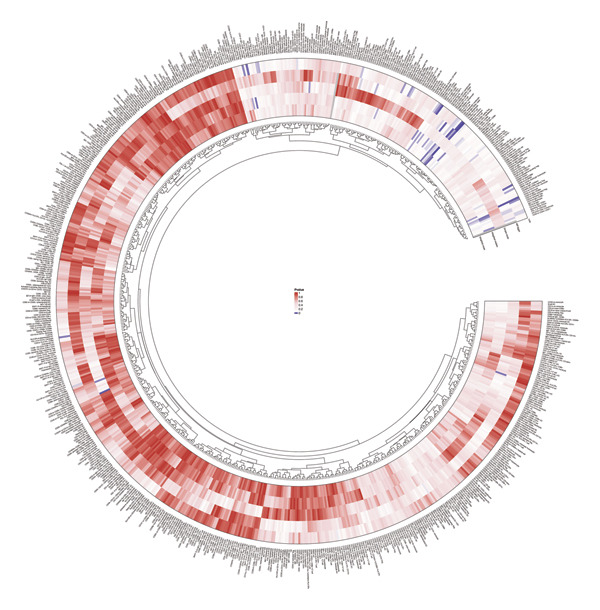
All *p* values of MR analysis between immune cells and RB1.

Based on the IVW MR analysis, we have determined that there is a causal relationship between the main result, RB1 protein, and 28 immune cells (with the threshold of *p* < 0.05). Our research revealed that CD3 on CD8br, FSC‐A on CD14+ monocyte, CD25 on CD39+ activated Treg, Mo MDSC AC, CD14− CD16+ monocyte %monocyte, CD19 on IgD + CD24−, Secreting Treg %CD4, CD45RA− CD4+ %CD4+, IgD− CD27− %B cell, and CD28 on secreting Treg all have a detrimental effect on RB1 protein. On the contrary, TCRgd %T cell, CD24 on IgD‐ CD38‐, HLA DR on DC, CD27 on IgD‐ CD38−, CD20 on IgD + CD38br, CD19 on CD24+ CD27+, CD20 on transitional, CD3‐lymphocyte %leukocyte, HLA DR on CD14+ monocyte, CD27 on IgD + CD38−unsw mem, TD CD4+ %T cell, CCR7 on naive CD8br, CD45RA on TD CD8br, CD45RA‐ CD28‐ CD8br %T cell, TCRgd %lymphocyte, BAFF‐R on IgD‐ CD38br, naive CD4+ %CD4+, and CD27 on sw mem are positively associated with RB ‐associated protein. We used forest plots and scatter plots to visually represent the correlation findings for exposures that exhibit a distinct causal link, respectively (Figures 2 and 3). Remarkably, the odds ratios for all the approaches consistently fall on one side of 1 for all the causal links examined, indicating a high level of homogeneity. The loop plots display the *p* values of all exposures for outcomes in color (Figure 4), while more information can be found in Supporting Table 2. The MR Egger, weighted mode, simple mode, and weighted median techniques produced comparable causal estimates in terms of both size and direction. Our analysis employing the MR Egger regression intercept technique did not reveal any indication of horizontal pleiotropy for gut microbiota in neurological diseases, with a *p* value > 0.05. The MR‐PRESSO analysis did not identify any outliers in the findings. Assuming there is no variation in characteristics and no multiple effects, the outcomes of IVW may be considered reliable. The pleiotropy and heterogeneity test findings are shown in Table 1. Furthermore, the leave‐one‐out analysis indicated that there were no significant variations in the estimated causal effects of any exposure to RB1 protein. This suggests that none of the detected causal relationships were influenced by any individual IV (Figure 5).

FIGURE 3The scatter plots of immune cells on RB1.

**Figure:**
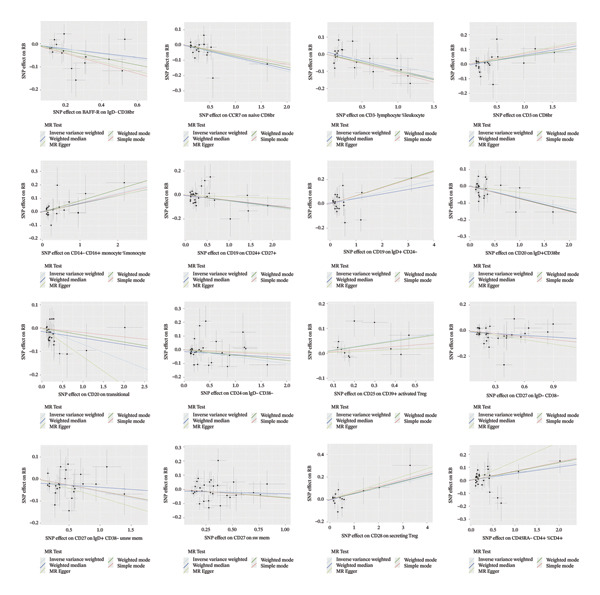
(a)

**Figure:**
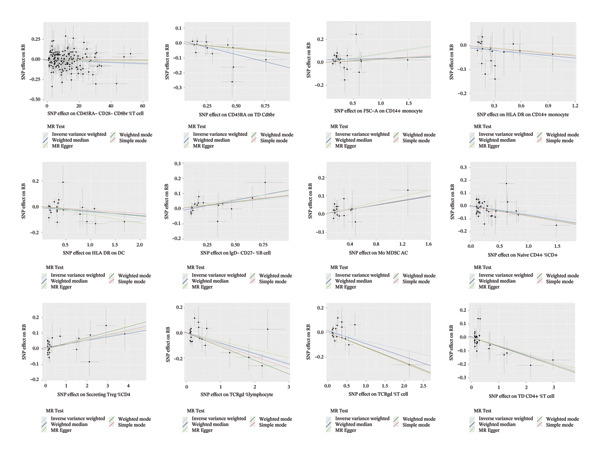
(b)

**TABLE 1:** Sensitivity analysis between exposures and RB1.

Outcome	Exposure	Types of exposure	MR Egger_heterogeneity_pval	IVW_heterogeneity_pval	egger_intercept	se	pleiotropy_pval	MR_PRESSO_pval
RB1	BAFF‐R on IgD‐CD38br	Immune cells	0.412421603	0.485865015	−0.00456119	0.019723305	0.820713681	0.426
RB1	CCR7 on naive CD8br	Immune cells	0.674727218	0.731013813	−0.002504418	0.014711535	0.866624977	0.715
RB1	CD3 on CD8br	Immune cells	0.806733621	0.830191084	−0.006750877	0.010960911	0.544277666	0.855
RB1	CD3‐lymphocyte %leukocyte	Immune cells	0.408487736	0.40900187	0.013597173	0.01366603	0.332943093	0.457
RB1	CD14‐CD16+ monocyte %monocyte	Immune cells	0.93655698	0.952887403	0.003887841	0.011466723	0.738102937	0.966
RB1	CD19 on CD24+ CD27+	Immune cells	0.722286276	0.759309091	−0.004033522	0.010169195	0.694639024	0.805
RB1	CD19 on IgD + CD24‐	Immune cells	0.545026438	0.580315519	0.006632037	0.010604134	0.537852251	0.58
RB1	CD20 on IgD + CD38br	Immune cells	0.690446716	0.737728859	0.003093413	0.010944193	0.779970733	0.729
RB1	CD20 on transitional	Immune cells	0.728415793	0.688124468	−0.014398125	0.011357844	0.218165397	0.581
RB1	CD24 on IgD‐ CD38‐	Immune cells	0.424187006	0.404951383	−0.013038726	0.011152772	0.252580913	0.429
RB1	CD25 on CD39+ activated Treg	Immune cells	0.338023119	0.426935399	−3.76659E‐05	0.027016684	0.998918026	0.461
RB1	CD27 on IgD‐ CD38‐	Immune cells	0.654530535	0.673467828	−0.009341475	0.012273018	0.452719148	0.709
RB1	CD27 on IgD + CD38− unsw mem	Immune cells	0.647877945	0.632206749	−0.01831509	0.016654338	0.282832487	0.671
RB1	CD27 on sw mem	Immune cells	0.449514523	0.474979671	−0.011561009	0.016005879	0.476101276	0.554
RB1	CD28 on secreting Treg	Immune cells	0.730625154	0.786624922	−0.003675488	0.012627166	0.77525963	0.81
RB1	CD45RA on TD CD8br	Immune cells	0.67822002	0.678307896	0.022007695	0.023159486	0.360723716	0.699
RB1	CD45RA‐CD4+ %CD4+	Immune cells	0.436286534	0.487220248	0.002713782	0.009923687	0.786656335	0.541
RB1	CD45RA‐CD28‐CD8br %T cell	Immune cells	0.519890981	0.531944681	−0.005827162	0.008486872	0.493375306	0.528
RB1	FSC‐A on CD14+ monocyte	Immune cells	0.55401554	0.439835229	0.023198582	0.013734364	0.10599654	0.43
RB1	HLA DR on CD14+ monocyte	Immune cells	0.672500738	0.715545716	−0.008160241	0.015870703	0.61338794	0.76
RB1	HLA DR on DC	Immune cells	0.094432856	0.108574463	−0.010390981	0.016251627	0.530628833	0.157
RB1	IgD‐ CD27‐%B cell	Immune cells	0.730176871	0.721280057	−0.016365504	0.016328161	0.333214764	0.784
RB1	Mo MDSC AC	Immune cells	0.869602137	0.906860101	0.0015231	0.018420612	0.935195654	0.925
RB1	Naive CD4+ %CD4+	Immune cells	0.327137711	0.363118139	0.004431591	0.010521791	0.676272823	0.394
RB1	Secreting Treg %CD4	Immune cells	0.459438625	0.46646052	0.008422983	0.008887183	0.354027562	0.534
RB1	TCRgd %lymphocyte	Immune cells	0.148561541	0.185148115	0.00252368	0.011537006	0.82918007	0.237
RB1	TCRgd %T cell	Immune cells	0.350199747	0.338546514	0.013892094	0.012936514	0.298811483	0.325
RB1	TD CD4+ %T cell	Immune cells	0.370717365	0.419874259	0.002682329	0.009050501	0.769393007	0.517

FIGURE 5Leave‐one‐out results of immune cells with a causal relationship to RB1.

**Figure:**
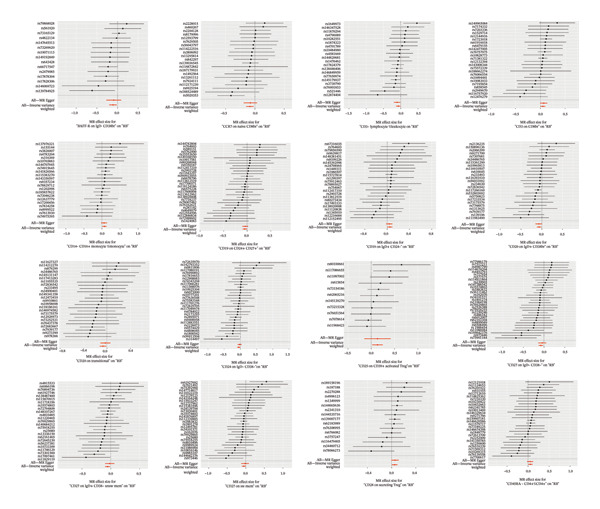
(a)

**Figure:**
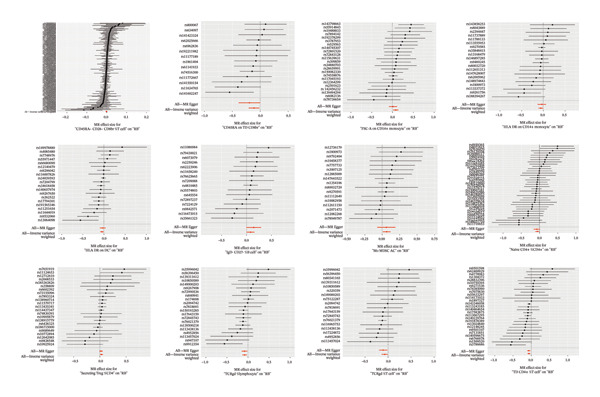
(b)

### 3.2. Single‐Cell Analysis Result

We conducted an analysis of scRNA‐seq data (GSE174202) obtained from four samples. These samples consisted of human retina tissue cells, with two samples derived from healthy individuals and two samples derived from individuals with RB. Following the use of rigorous quality control criteria to preprocess the data, we employed the UMAP approach to display the high‐dimensional scRNA‐seq data. This visualization was based on the top 15 main components. Afterward, we effectively categorized the cells into 14 subclusters and identified their recognized cell types using the SingleR program. The main cellular components were neuroepithelial cells, neurons, endothelial cells, and astrocytes (Figure 6). Our analysis revealed that neuroepithelial cells and neurons were the two most abundant cell types in RB tissues. Consequently, we conducted independent classifications and analyses for each of their subsets. Plasmacytoid dendritic cells, plasmablasts, and progenitor cells are included in neuroepithelial cell; progenitor cells and plasmablasts are discovered in the RB issue, and naive T cells (CD8+) as cell subset of neurons are discovered in the healthy controls. We used UMAP and DIMplot techniques to visually represent the distribution and percentage of individual cell subpopulations (Figure 7). ScRNA‐seq allows for the acquisition of cells at different stages, allowing the investigation of uninterrupted differentiation paths. The UMAP dimensionality reduction approach surpasses other algorithms in accurately capturing the overall and topological structures of datasets, including the spatial arrangement of individual cells. We used the slingshot *R* package to evaluate cellular differentiation processes, as shown in Figures 8(a) and 8(b). The CellChat algorithm detected the presence of ligand‐receptor pairings (MDK‐SDC2) among the primary cell types found in the cavernous tissue milieu, as shown in Figures 8(c), 8(d), 8(e), and 8(f).

FIGURE 6Single‐cell transcriptomics landscape of various samples. (a) The results of automated classification are depicted, showing various cell populations. (b) Proportion of each cell subset in different samples.

**Figure:**
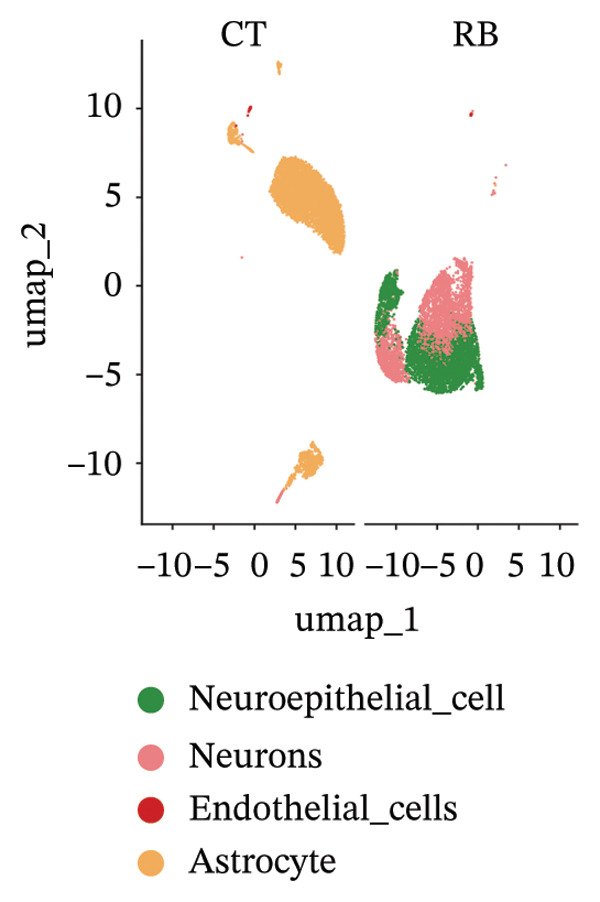
(a)

**Figure:**
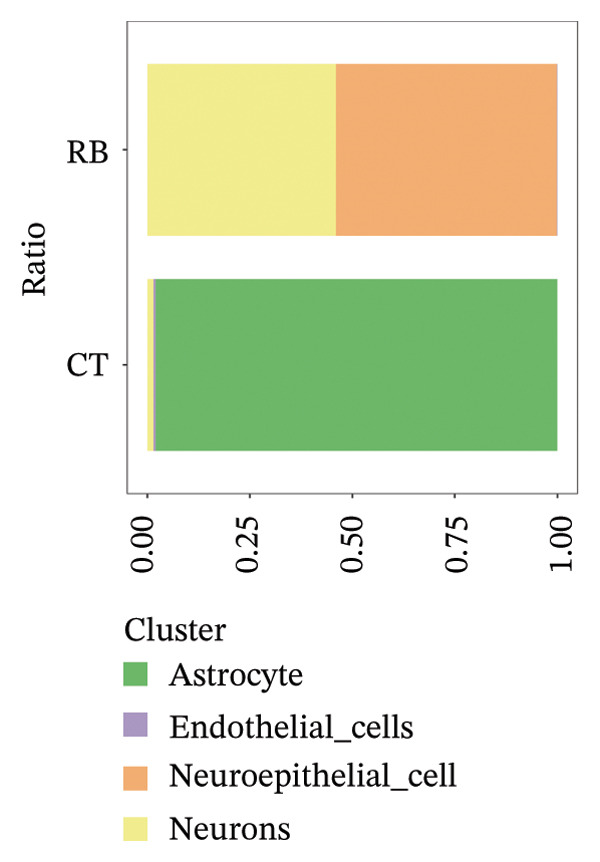
(b)

FIGURE 7(a and b) Classification of cell subsets of Neuroepithelial_cell. (c and d) Classification of cell subsets of neurons.

**Figure:**
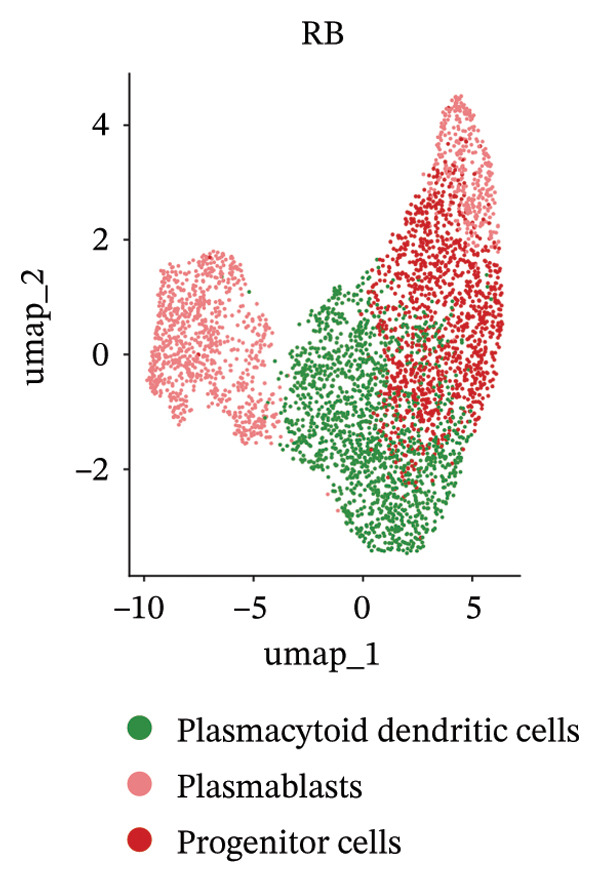
(a)

**Figure:**
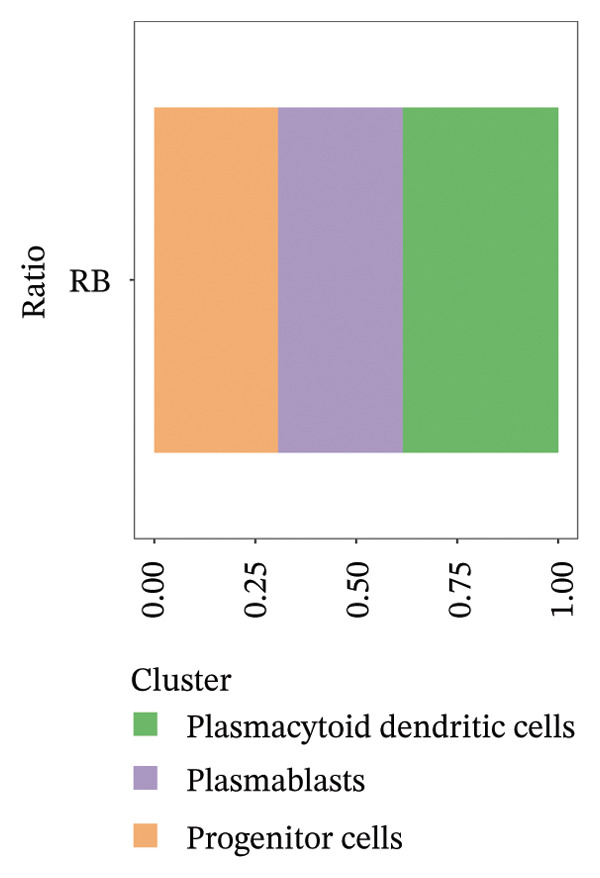
(b)

**Figure:**
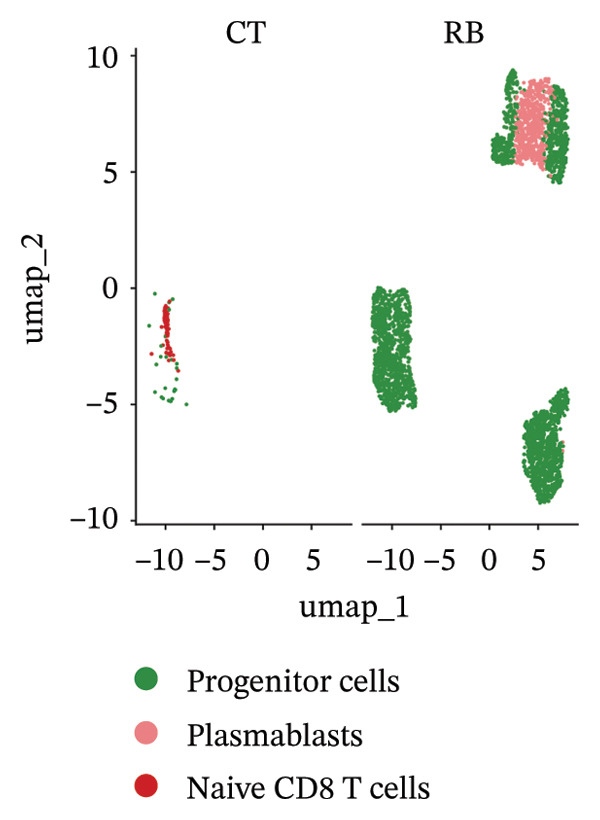
(c)

**Figure:**
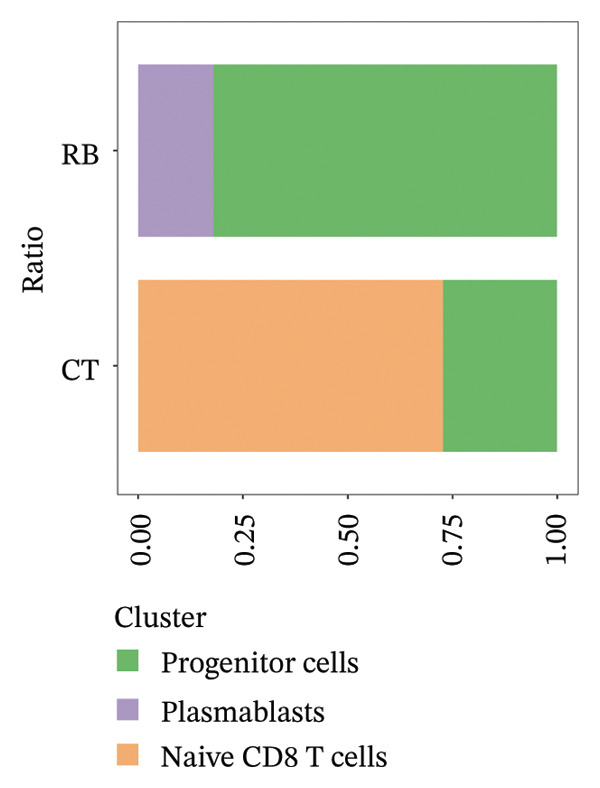
(d)

FIGURE 8(a) Trajectory analysis of Neuroepithelial_cell subset. (b) Trajectory analysis of neuron subset. (c and d) Cell communication between plasmacytoid dendritic cells and other cells. (e and f) Cell communication between progenitor cells and other cells.

**Figure:**
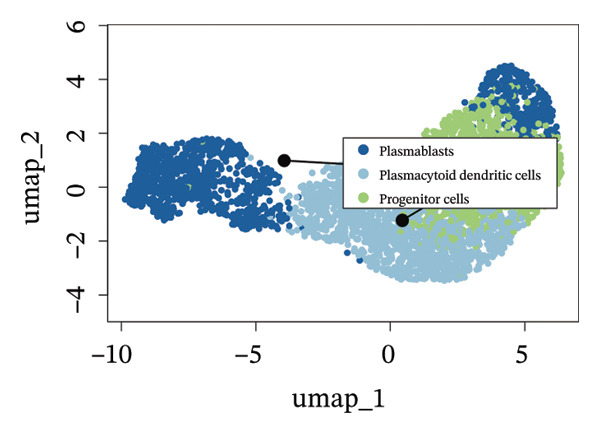
(a)

**Figure:**
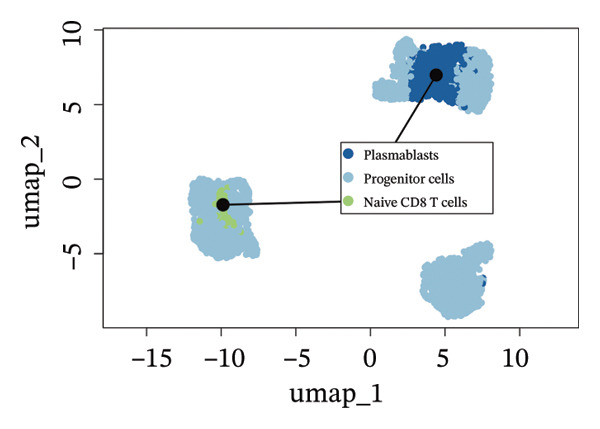
(b)

**Figure:**
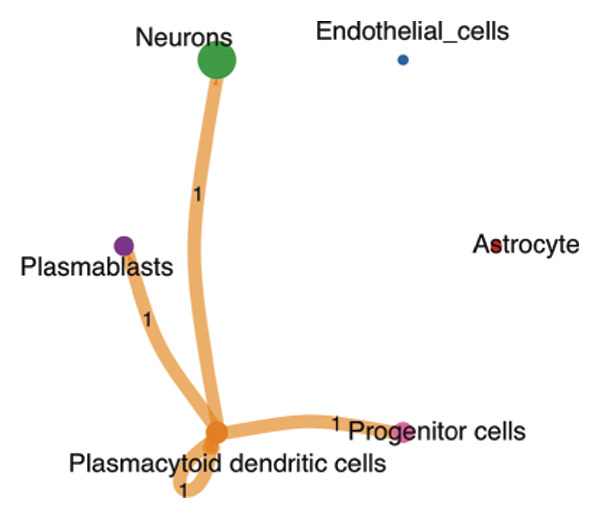
(c)

**Figure:**
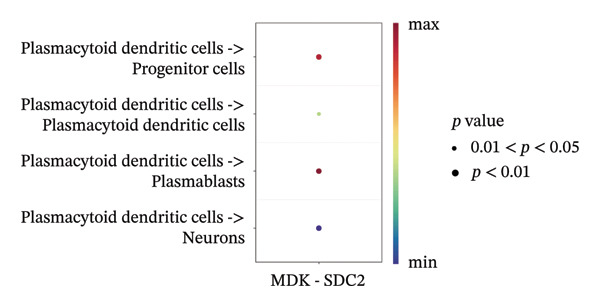
(d)

**Figure:**
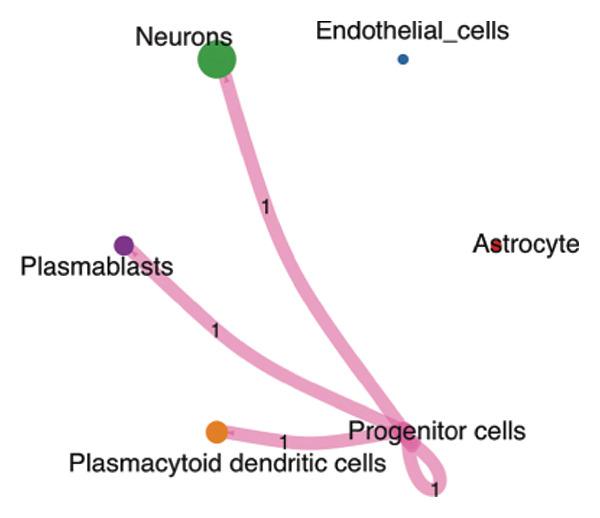
(e)

**Figure:**
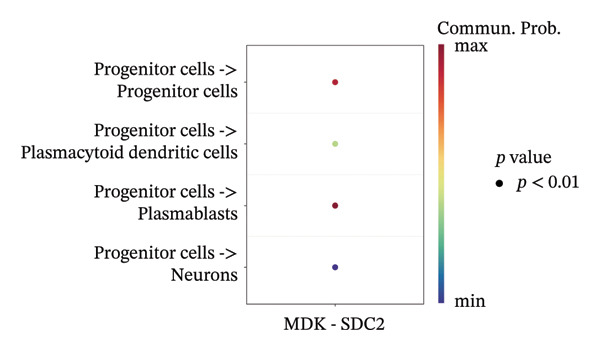
(f)

### 3.3. MR Analysis Using Key Marker Genes Identifies Novel Causal Genes That Can Mediate the Effect of Exposure Factors on the Development of RB

We used MR analysis on scRNA‐seq data for the RB1 protein to identify crucial genes that may have an impact on RB. Using the Seurat tool, we first discovered specific genes that differentiate each cell fraction from other cell kinds and subtypes. Additionally, we selected relevant indicators for additional investigation. In order to further investigate, we effectively transformed gene symbols into their matching ENSEMBL IDs utilizing the org.Hs.eg.db database. Subsequently, a two‐sample MR study was conducted. This analysis included obtaining SNP data associated with our genes of interest as the exposure data, getting lung adenocarcinoma outcome data from the EBI database, and ensuring consistency between the exposure and outcome datasets. The MR study identified a number of genes that are strongly linked to the RB1 protein. A volcano plot was created to graphically display the relationship between the ‐log10‐transformed *p* values and ln (OR) for each gene. Genes with significant *p* values were highlighted in the figure (Figure 9). The plot clearly identifies genes that have strong positive and negative relationships. Afterward, we created a forest plot to display the OR and 95% CI for each significant gene. This plot highlights the strength and direction of the connection for each gene (Figure 10). Subsequently, we conducted a search for these genes within the SNPs related to exposure factors. Our investigation revealed that EZH2 (rs34900401), UBCLP1 (rs146974582), and HKDC1 (rs148384064) were the pivotal genes that exhibited SNPs of CD20 on transitional and CD45RA‐ CD28‐ CD8br %T cell and had an influence on the result of the RB1 protein.

FIGURE 9Neuroepithelial_cell subset: (a) A volcano plot showing a causal relationship between key genes and RB1 in plasmacytoid dendritic cells. (b) A volcano plot showing a causal relationship between key genes and RB1 in plasmablasts. (c) A volcano plot showing a causal relationship between key genes and RB1 in progenitor cells. Neuron subset: (d) A volcano plot showing a causal relationship between key genes and RB1 in naive CD8 T cells. (e) A volcano plot showing a causal relationship between key genes and RB1 in plasmablasts. (f) A volcano plot showing a causal relationship between key genes and RB1 in plasmablasts.

**Figure:**
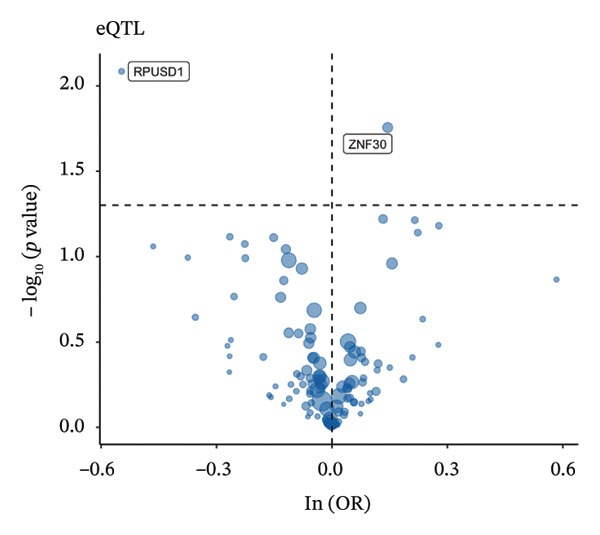
(a)

**Figure:**
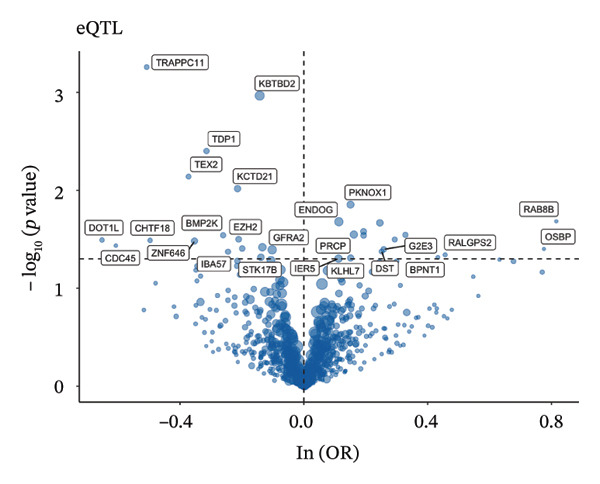
(b)

**Figure:**
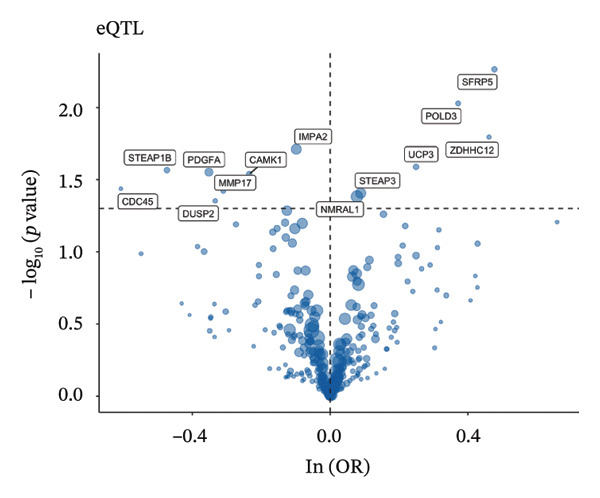
(c)

**Figure:**
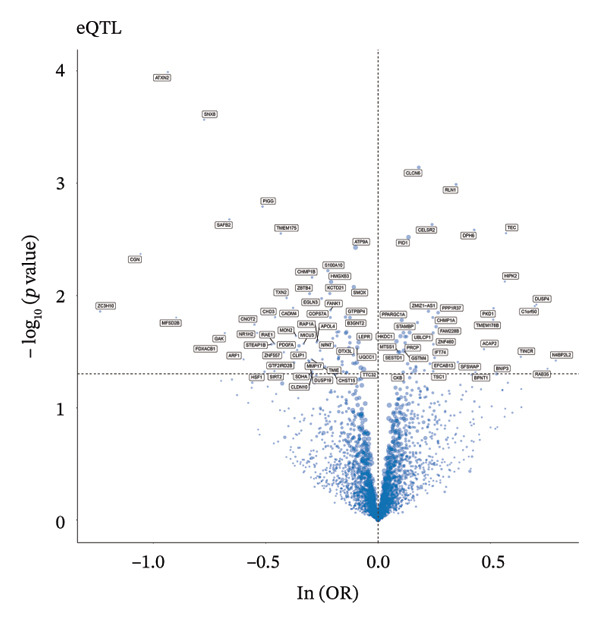
(d)

**Figure:**
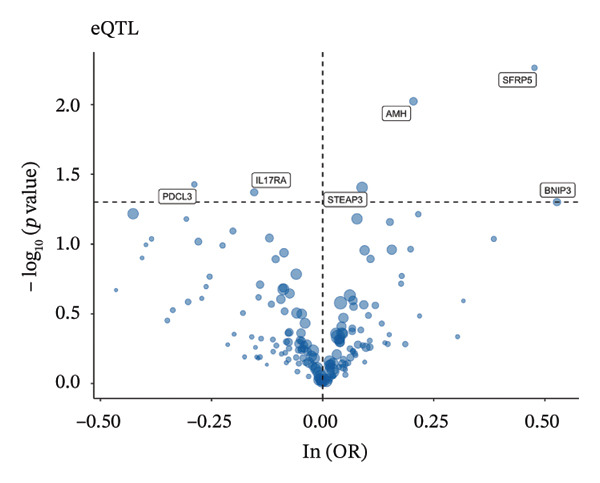
(e)

**Figure:**
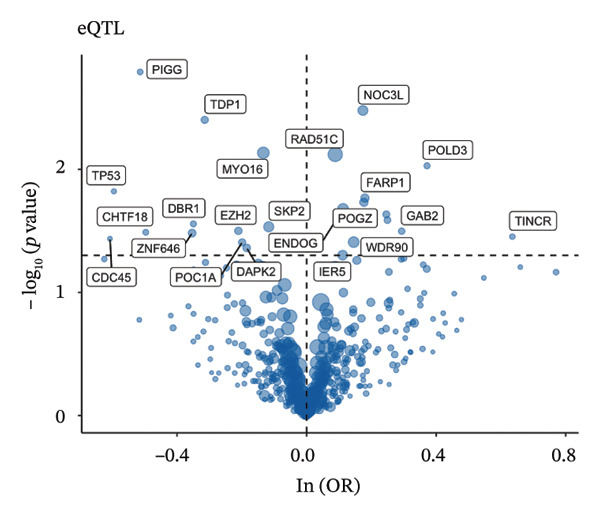
(f)

FIGURE 10Neuroepithelial_cell subset: (a) A forest plot showing a causal relationship between key genes and RB1 in plasmacytoid dendritic cells. (b) A forest plot showing a causal relationship between key genes and RB1 in plasmablasts. (c) A forest plot showing a causal relationship between key genes and RB1 in progenitor cells. Neuron subset: (d) A forest plot showing a causal relationship between key genes and RB1 in naive CD8 T cells. (e) A forest plot showing a causal relationship between key genes and RB1 in plasmablasts. (f) A forest plot showing a causal relationship between key genes and RB1 in plasmablasts.

**Figure:**
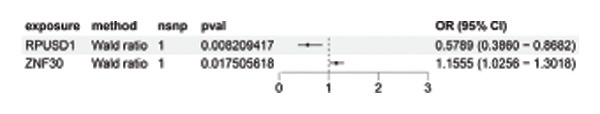
(a)

**Figure:**
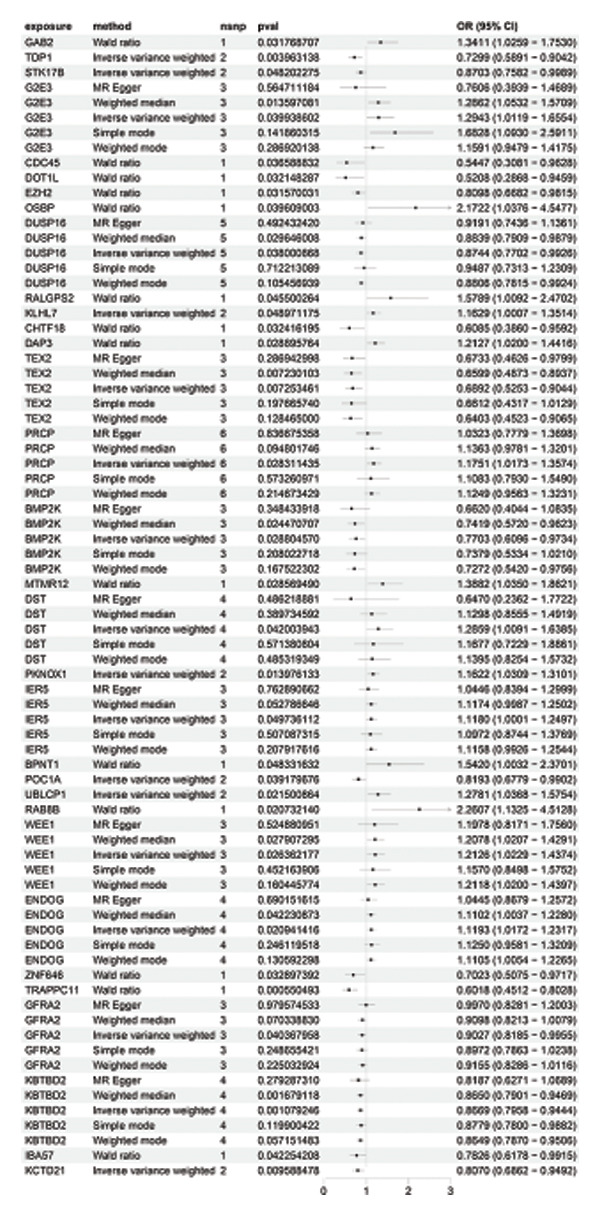
(b)

**Figure:**
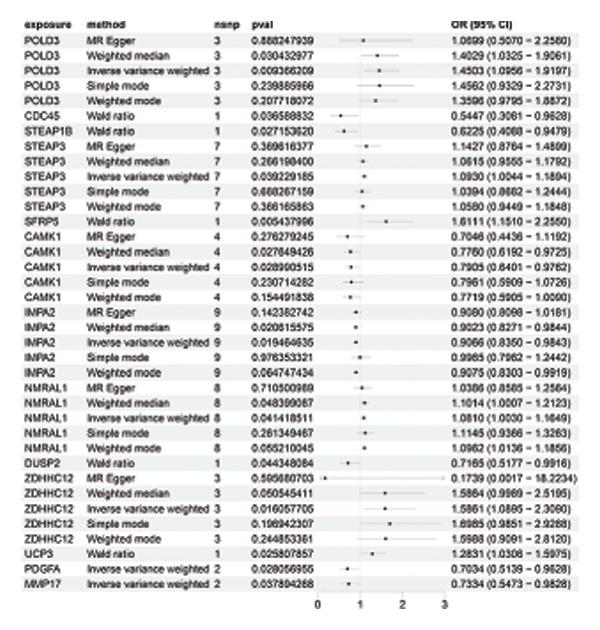
(c)

**Figure:**
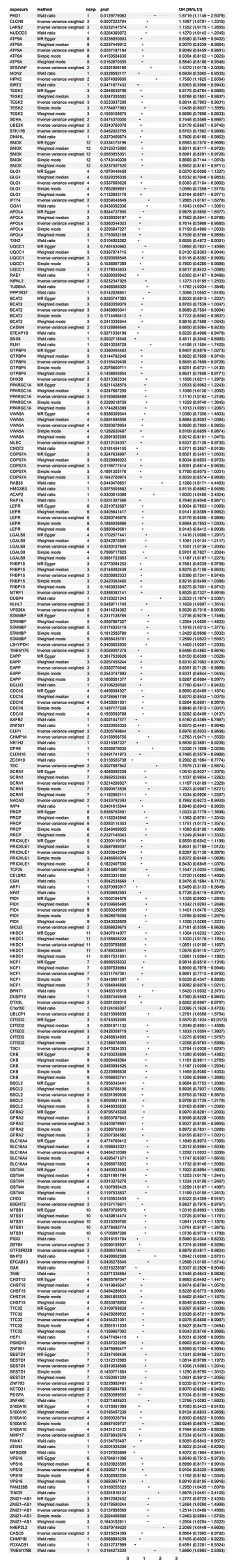
(d)

**Figure:**
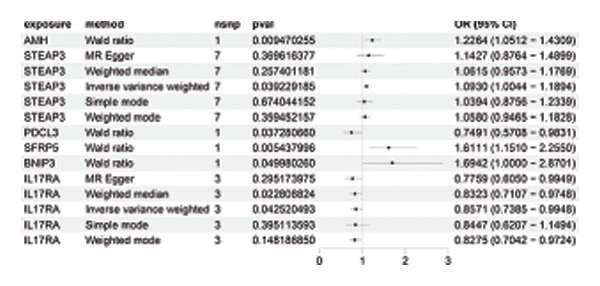
(e)

**Figure:**
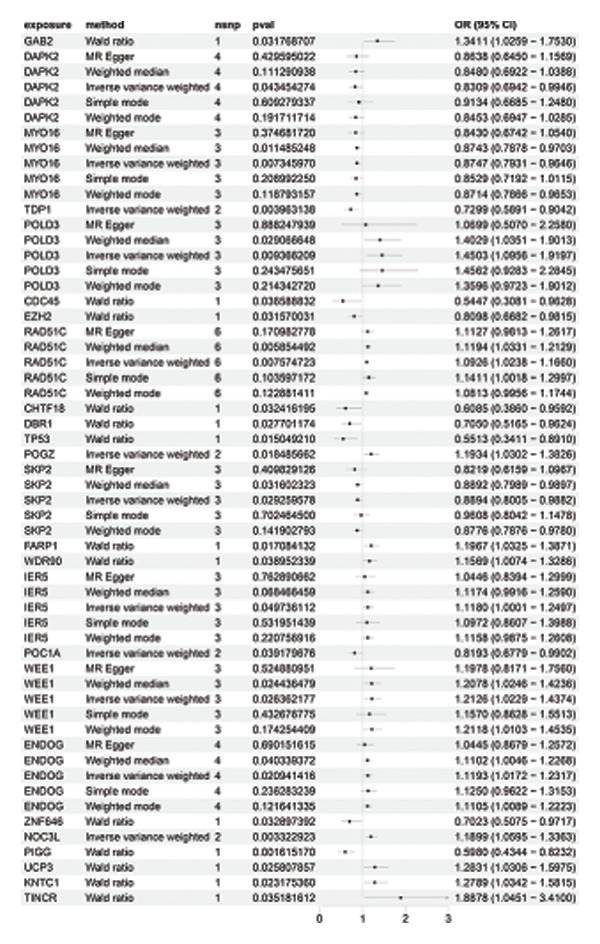
(f)

### 3.4. Single‐Cell Transcriptomic Analysis Reveals EZH2 and UBCLP1’s Crucial Role in Metabolism and Function in RB

We first visualized the expression patterns of key marker genes including EZH2 and UBCLP1 across various cell clusters within Neuroepithelial_cell (Figures 11(a) and 11(b)). Figures 11(c) and 11(d) illustrate the variations in key genes, particularly the expression of EZH2, among several subtypes of neurons. Remarkably, UBCLP1 and HKDC1 were shown to have a causal relationship with RB1 proteins in naive CD8 T cells. Nevertheless, the limited expression of UBCLP1 and HKDC1 in naive CD8 T cells resulted in the absence of substantial differences in cellular communication and metabolic pathways. In order to examine changes in gene expression throughout cell growth or transcriptional dynamics, we represented the activated or deactivated states of different genes over pseudotime (as shown in Figures 12(a) and 12(b)). The link between the expression of genes EZH2 and UBCLP1 and pseudotime was shown using scatter plot analysis (Figures 13(a), 13(b), and 13(c)). The statistical analysis of Figure 13(a) revealed a modest positive association (Pearson *r* = 0.13, *p* < 0.001), indicating a possible rise in EZH2 expression in progenitor cells as pseudotime progresses. In contrast, the expression of EZH2 may decrease in plasmablasts as pseudotime progresses, as seen in Figure 13(b) (Pearson *r* = −0.1, *p* < 0.001). UBCLP1 did not provide any significant results, and the causal links can be shown in Figure 13(c). Figure 14 illustrates the differences in metabolic pathways between cells that are negative and positive for certain genes. In EZH2+ progenitor cells, the retinol metabolism, porphyrin and chlorophyll metabolism, and pantothenate and CoA biosynthesis pathways were shown to be impaired.

FIGURE 11(a) Expression distribution of the EZH2 gene across cell clusters of Neuroepithelial_cell plotted using UMAP. (b) Expression distribution of the UBLCP1 gene across cell clusters of Neuroepithelial_cell plotted using UMAP. (c) Expression distribution of the EZH2 gene across cell clusters of neurons plotted using UMAP. (d) Expression pattern of key genes across different cell subpopulations.

**Figure:**
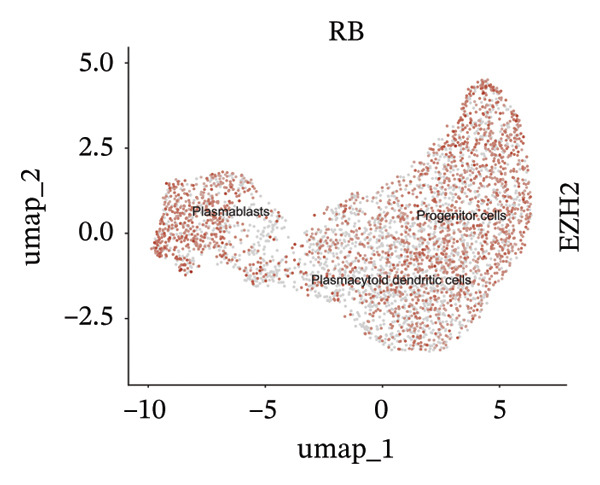
(a)

**Figure:**
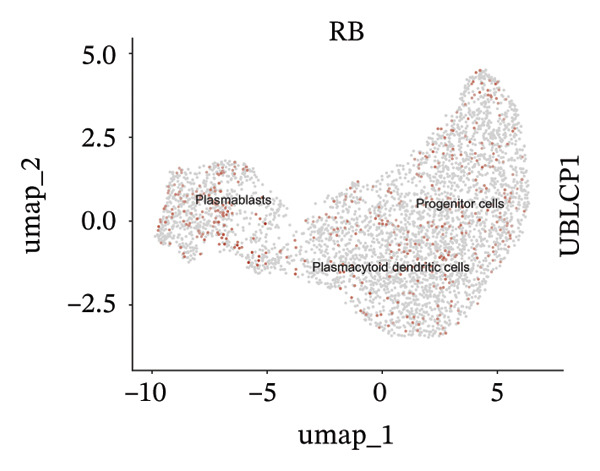
(b)

**Figure:**
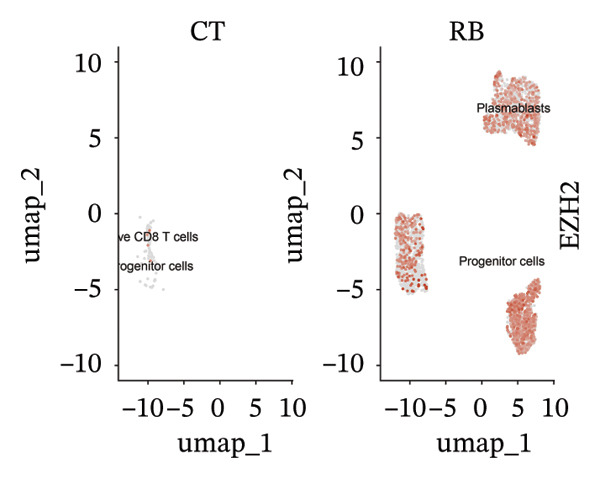
(c)

**Figure:**
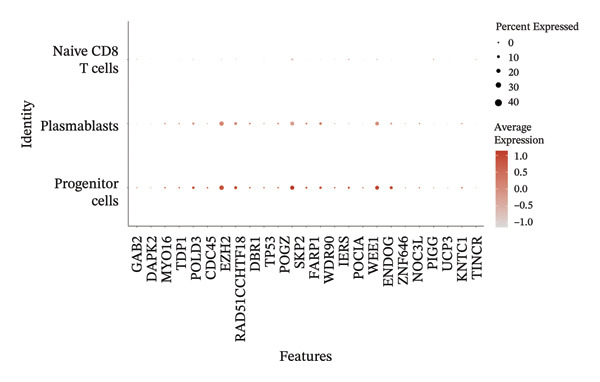
(d)

FIGURE 12(a) Gene on/off status in pseudotime, reflecting gene expression dynamics of plasmablasts during cellular developmental or transcriptional processes. (b) Gene on/off status in pseudotime, reflecting gene expression dynamics of progenitor cells during cellular developmental or transcriptional processes.

**Figure:**
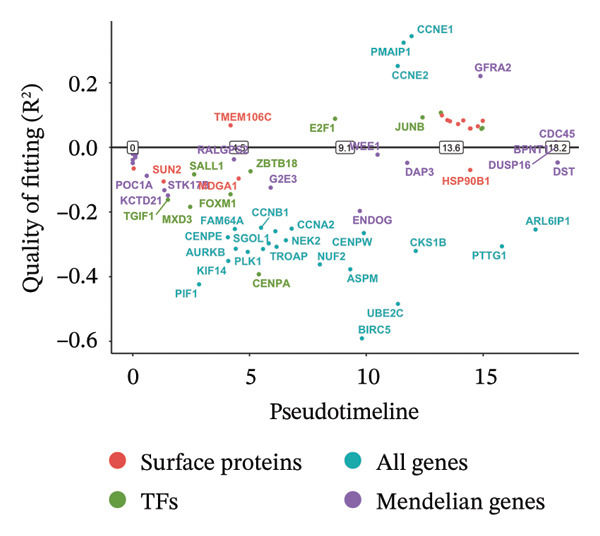
(a)

**Figure:**
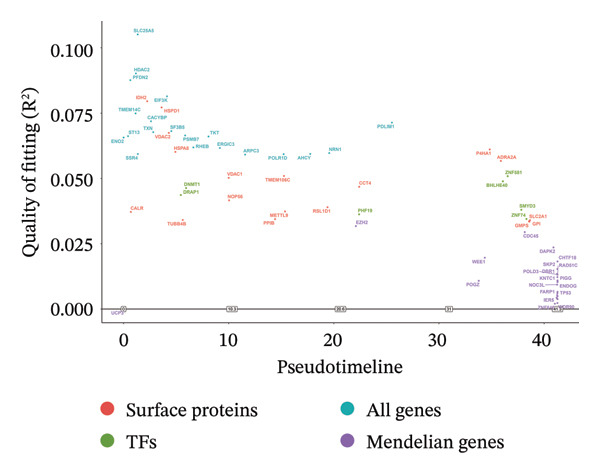
(b)

FIGURE 13(a) Scatter plot depicting the relationship between the expression of the EZH2 gene and pseudotime in progenitor cells. (b) Scatter plot depicting the relationship between the expression of the EZH2 gene and pseudotime in plasmablasts. (c) Scatter plot depicting the relationship between the expression of the UBCLP1 gene and pseudotime in plasmablasts.

**Figure:**
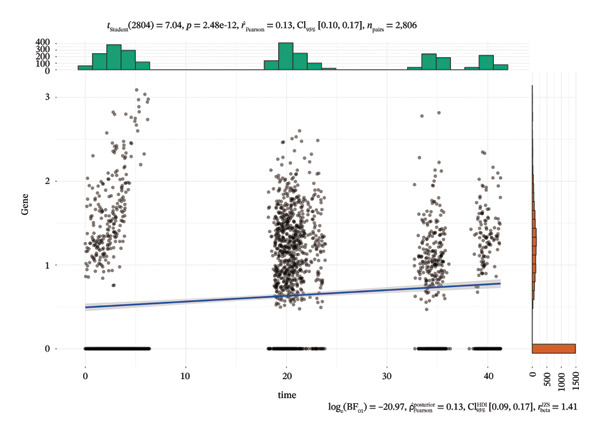
(a)

**Figure:**
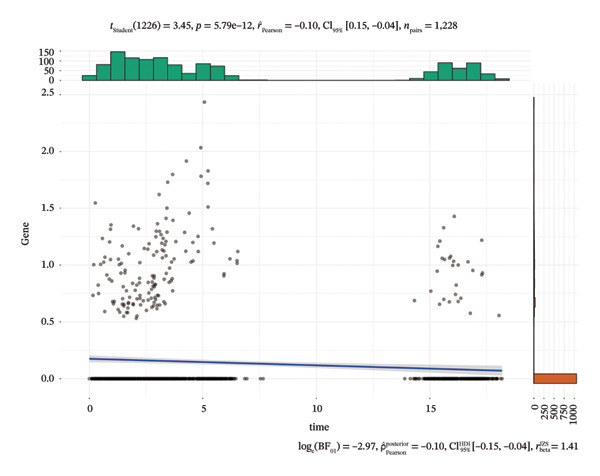
(b)

**Figure:**
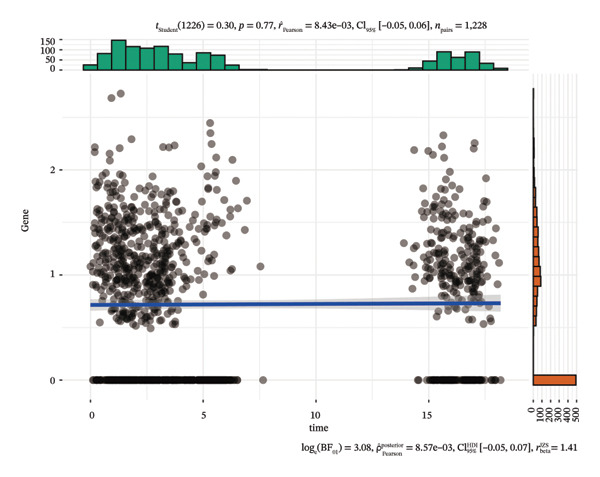
(c)

FIGURE 14(a) Metabolic pathway analysis showing the activation states, revealing differences in metabolic activities between EZH2 +  plasmablasts and EZH2‐plasmablast cell subgroups. (b) Metabolic pathway analysis showing the activation states, revealing differences in metabolic activities between UBCLP1 +   plasmablast and UBCLP1‐plasmablast cell subgroups. (c) Metabolic pathway analysis showing the activation states, revealing differences in metabolic activities between EZH2 +   progenitor cells and EZH2‐progenitor cell subgroups.

**Figure:**
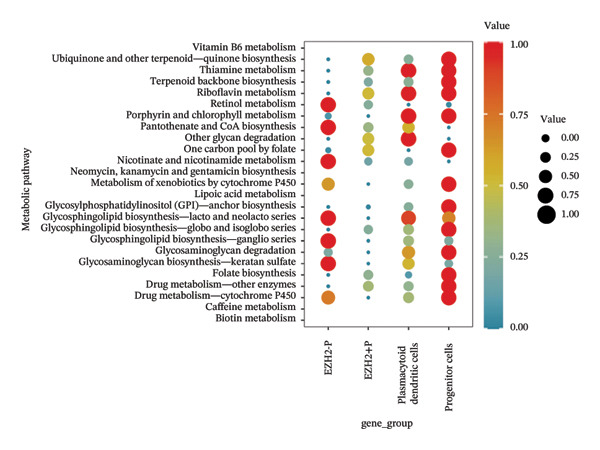
(a)

**Figure:**
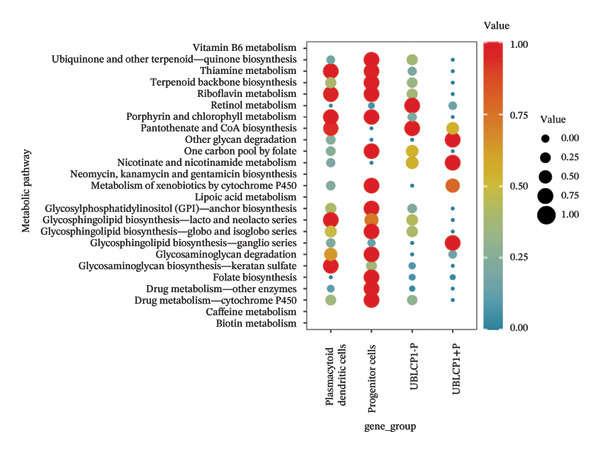
(b)

**Figure:**
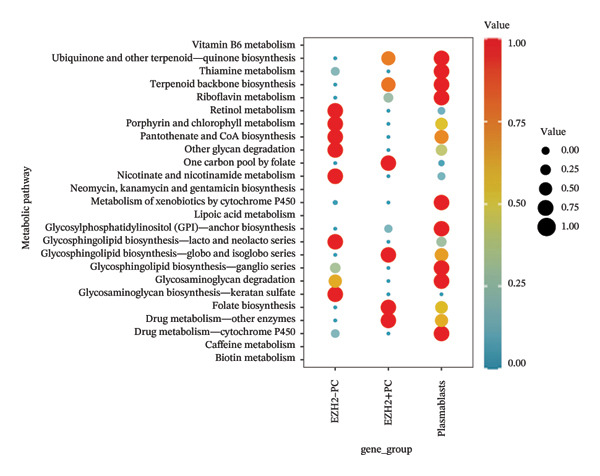
(c)

## 4. Discussion

In this work, we did a two‐sample MR analysis utilizing summary statistics data from the largest GWAS of circulating immune cells. The GWAS was done by the MiBioGen Consortium and focused on the RB1 protein. The objective was to assess the causal correlation between immune cells and RB. Regrettably, the precise causative mechanism cannot be determined using MR analysis. Therefore, we used the RB1 protein, which is strongly linked to the development of RB, as the factor of exposure. Furthermore, the analysis of single‐cell sequencing data revealed a direct correlation with RB1 genes. Specifically, single‐cell sequencing data mining also found a causal relationship with RB1 genes, among which EZH2, UBCLP1, and HKDC1 were identified as exposure SNPs, indicating that exposure factors may influence the onset and progression of RB by modulating the expression of these three genes.

RB tumor cells that develop at an early stage have a strong immune gene expression pattern, which is then accompanied by the buildup of dendritic, monocyte, macrophage, and T‐lymphocyte cells inside the RB tumors [20]. Early‐onset RB is more likely to be linked to abnormalities in the RB1 gene since these mutations tend to arise specifically during early fetal development. Furthermore, family RB often manifests at a young age, suggesting that genetic abnormalities in the RB1 gene have a significant influence on the occurrence of early‐onset instances. Hence, there is a strong correlation between immunophenotypes and the development of RB. Our investigation identified 28 potential causal connections between immunophenotypes and the protein linked with RB. Through the examination of the scRNA sequencing data, we have discovered key genes that may be influenced by the immunophenotype in the progression of RB.

In tumor tissues, the presence of CD3+ T cells and CD8+ T cells is considered to be a good prognostic indicator. In particular, tumor‐infiltrating CD8+ T cells (also known as cytotoxic T cells) recognize and kill tumor cells. In fact, several cancer treatments, such checkpoint inhibitors, work against tumors by making these CD8+ T cells more active. A study conducted on patients with metastatic melanoma revealed a favorable correlation between the presence of CD3+ and CD8+ T cells inside tumors and the overall survival and disease‐free survival of patients [21]. Researchers reached similar findings in another study conducted on people with colorectal cancer [22]. This notion has been validated in other kinds of malignancies, such as lung, bowel, stomach, and breast cancers. Therefore, our work has shown that CD3 on CD8br is a significant protective factor in a considerable number of cancers. Additionally, we have found a positive correlation between CD3 on CD8br and RB1, suggesting that CD3 on CD8br may also serve as a protective factor for RB. The decrease in CD247 expression was significantly linked to the presence of CD3 on CD8br cells in RB.

Our investigation revealed that the presence of CD27 on IgD −CD38 − , CD27 on IgD + CD38− unswitched memory cells, and CD27 on switched memory cells are associated with an increased risk of RB. Additionally, we discovered that CD27, a molecule strongly associated with these three immunological characteristics, was increased in RB1‐deficient RB tissues. CD27, a member of the TNF receptor superfamily, is extensively present in fully developed T and B cells, and its primary function is to augment cell activation, cell viability, and the production of memory cells. There is no doubt that this has a suppressive impact on cancers. However, conversely, some studies indicates an elevated presence of CD70 (the ligand for CD27) and proposes that this might possibly serve as a means for tumors to exploit the CD70‐CD27 pathway in order to control immune cells that infiltrate the tumor, thereby facilitating immune evasion [23]. As liver cancer progresses, the cancer cells in the liver may produce CD27, a protein that helps them avoid being attacked by the immune system. This allows the cancer cells to proliferate and spread more easily. Furthermore, it has been suggested that cells of nasopharyngeal carcinoma can enhance the formation and function of regulatory T cells (a specific type of immune‐suppressing cell) by means of the interaction between CD70 and CD27. This interaction creates an immunosuppressive setting, which, in turn, promotes the progression of nasopharyngeal carcinoma [24].

Furthermore, studies have shown that some cancers might induce immune evasion by activating B cells, particularly CD27+ B cells. Upon encountering tumor cells, some B cells have the potential to undergo a transformation into regulatory B cells that contain the ability to suppress the immune system, hence hindering the assault on tumors by other immune cells. Our results suggested that RB cells may also exhibit immune evasion by overexpressing CD70 to induce the activation of CD27+B cells. Of course, this result still needs further experiments and clinical evidence.

What is more, the investigation revealed two intriguing immunological phenotypes: CD20 on IgD + CD38br and CD20 on transitional. CD20 is a cell surface antigen that is mostly found on B cells. From the pro‐B cell phase forward, the expression of this gene increases gradually as maturation takes place. Playing a critical role in activating B cells for immunological responses, it is an important target for therapeutic interventions in several B cell illnesses, including lymphomas and leukemia.

We are pleased to see that our findings from analyzing single‐cell sequencing data align with our MR results. The EZH2 and UBLCP1 genes were identified as IVs for CD20 on transitional. The EZH2 gene was shown to be a risk factor for the RB1 protein, while the UBLCP1 gene was found to be a protective factor. These two genes are crucial genes that have been identified as being strongly associated with the progression of RB. EZH2, also known as enhancer of zeste homolog 2, is an important epigenetic modifier. Its primary role is to silence gene transcription by adding methyl groups to the H3K27 locus of chromatin histones, resulting in trimethylation modification. Multiple studies have extensively shown its strong correlation with RB. Qifang Jin and his colleagues discovered that microRNA‐101‐3p inhibits the growth of RB cells by specifically targeting EZH2 [25]. Specific inhibition of EZH2 may selectively compromise the viability of RB cells while leaving normal cells unaffected, offering a novel approach for RB therapy. In our work, we conducted a more in‐depth examination of the gene’s expression and discovered that it was present in both progenitor cells (a subtype of neurons) and plasmablast cells (a subtype of Neuroepithelial_cells). Furthermore, the level of expression in these cells was much greater than in other cell types. Nevertheless, the level of EZH2 expression differed between these two cell lines. Significantly, after leaving the bone marrow and moving to the periphery, transitional B cells come across antigens, undergo additional maturation, and transform into plasmablasts that are capable of generating antibodies. Transitional B cells and plasmablasts have a close connection, and EZH2 plays a vital role in maintaining this connection. We identified modifications in 25 metabolic pathways in EZH2‐positive plasmablast cells, which include retinol metabolism, pantothenate and CoA production, and glycosaminoglycan biosynthesis—specifically keratan sulfate. The discoveries have significant worth in the management of RB and the advancement of medications that focus on EZH2. On the other hand, UBLCP1 and HKDC1 are expressed in naive CD8 T cells and are causally related to RB1. The RB1 protein, as a key regulator of the cell cycle, may have its stability and activity influenced by the process of ubiquitination, particularly through the ubiquitination mediated by E3 ubiquitin ligases such as the CRL4 complex, which could lead to the degradation of RB1 [26]. On the other hand, UBLCP1, as a proteasome phosphatase, regulates the activity of the 26S proteasome by dephosphorylating the Rpt1 subunit, affecting the degradation process of proteins [27]. Therefore, RB1 may be marked for degradation through the ubiquitination pathway, and UBLCP1 affects this degradation process by regulating the activity of the proteasome. If RB1 is one of the substrates of the proteasome activity regulated by UBLCP1, then changes in the activity of UBLCP1 may affect the degradation rate of RB1, thereby influencing the regulation of the cell cycle. This interaction may play a role in tumorigenesis and the control of cell proliferation [28], but further experimental research is needed to verify this predicted relationship. HKDC1 may regulate glycolysis and energy metabolism [29, 30], thereby affecting the proliferation status of cells and indirectly influencing the function of RB1. Since RB1 plays a central role in regulating the cell cycle and inhibiting excessive cell proliferation, any factor that affects cellular metabolism and energy status may indirectly affect the activity of RB1 and the progression of the cell cycle. Therefore, HKDC1 may indirectly regulate the function of RB1 by altering the metabolic state of cells, affecting the control of the cell cycle and tumor suppression.

Although harmony integration successfully minimized technical batch effects between datasets (Figure 6), we acknowledge three inherent constraints: (1) Harmony assumes that batch effects are orthogonal to biological variation, which may not hold if technical artifacts correlate with disease states, (2) overcorrection risks attenuating subtle but biologically relevant signals, particularly in rare cell populations, and (3) residual confounding may persist due to fundamental protocol differences between stem cell‐derived and patient‐derived samples. To mitigate these limitations, we employed stringent quality controls and validated biological consistency through marker gene expression patterns. Future studies using uniformly processed samples would strengthen batch effect robustness.

Our study, by leveraging the summary statistics data from the largest circulating immune cell GWAS for two‐sample MR analysis, has for the first time revealed a causal association between the RB1 and immune cells. Additionally, through single‐cell sequencing analysis, we found that EZH2, UBLCP1, and HKDC1 as SNPs of exposure factors may regulate RB1, influencing the onset and progression of RB. The study also uncovered cellular‐level changes and functions of these genes. The results not only emphasize the strong correlation between immunophenotypes and the development of RB but also provide a new perspective for understanding the complex interactions in the tumor immune microenvironment. This is of significant importance for developing new therapeutic strategies and prognostic biomarkers.

## Funding

This research was supported by the Key Research and Development Technology project of Anhui Province (Grant No. 2022j11020013), the Natural Science Foundation of Tibet Autonomous Region (Grant No. XZ202501ZR0090), the Natural Science Foundation for Group‐Style Medical Aid Project of Tibet Autonomous Region (Grant No. XZZR202402034 [W]), and the College Students’ Innovation and Entrepreneurship Training Project of Anhui Province (Grant No. S202410366013).

## Ethics Statement

The authors have nothing to report.

## Consent

The authors have nothing to report.

## Conflicts of Interest

The authors declare no conflicts of interest.

## Supporting Information

Supporting 1. Supporting Table 1. IVs related to immune cells and RB1.

Supporting 2. Supporting Table 2. Casual effects of MR analysis between immune cells and RB1.

## Supporting information


**Supporting Information** Additional supporting information can be found online in the Supporting Information section.

## Data Availability

All data generated or analyzed during this study are included in this published article and its supporting information files. Our original data come from the public databases mentioned in this article.
